# PReS-FINAL-2301: Cardiac tamponade and auto-immune haemolysis as first presentation of JSLE

**DOI:** 10.1186/1546-0096-11-S2-P291

**Published:** 2013-12-05

**Authors:** M Yiallourides, J Shipman, S Rangaraj

**Affiliations:** 1 Paediatric Rheumatology, Nottingham University Hospitals, Nottingham, UK

## Introduction

This is a case report of a 14 year old girl who presented with cardiac tamponade and severe auto-immune Haemolytic anaemia at first presentation of Juvenile Systemic Lupus Erythematosus (JSLE).

## Objectives

This case highlights life-threatening presentations of JSLE.

## Methods

The case was derived from a retrospective review of case notes and laboratory findings. The patient was managed at a tertiary paediatric rheumatology centre and at a regional cardiothoracic unit. All authors have been involved in the medical management of the patient

## Results

### History

This previously well 14 year old girl has been under the care of her primary care physician for a period of five months with history of general malaise, lethargy, poor academic performance and arthralgia particularly of her fingers and ankles. Her symptoms were preceded by an upper respiratory tract infection. One month prior to her admission she presented to the Emergency Department with chest pain and shortness of breath. She was diagnosed with a chest infection and treated with antibiotics. Over the next few weeks she continued to progressively get more tired with intermittent chest pains and migratory joint swellings. There was no history of rash. She developed fever and worsening breathlessness on lying down just before she was referred to secondary care.

### Examination

On examination she was pale, febrile, tachycardic, with mild respiratory distress. Cardiothoracic examination revealed dullness and reduced air entry on both lung bases, raised jugular venous pressure, pulsus paradoxus and muffled heart sounds. There was mild swelling of the right 5th MCP and PIP joints and mild left wrist restriction. There was no malar rash or other mucocutaneous lesions.

### Laboratory findings

A Chest X-ray and Echocardiography confirmed a large pericardial effusion with marked right atrium dysfunction. She also had bilateral pleural effusions. A full blood count and blood film was suggestive of severe autoimmune haemolyis (Hgb 50 g/L, IAT positive, DAT positive anti-IgG, anti-IgM, anti-C3d). Her ESR was high 181 mm/hr and CRP was mildly raised 13 mg/L. Her ANA titre was 400, anti-dsDNA>200 iu/mL with positive Clitheria test and positive anti-Ro, anti-Ro52 and antiphospholipid antibodies. ASOT serology and anti-Dnase B Streptococcal were negative.

## Treatment

Patient was transferred to a cardiothoracic unit for urgent pericardial drain insertion. 1200 mls of clear serous fluid were drained. She required two units of cross-matched blood peri-operative. She was initially treated with high dose oral steroids by the haematologist. Once her ANA result was known she was commenced on IV Methyl Prednisolone and two weekly cycles of Cyclophosphamide with good response.

## Conclusion

Pericardial effusion and cardiac tamponade is extremely rare as a first presentation of JSLE with only a few cases reported[1]. Early recognition of JSLE and aggressive treatment is vital to minimise the morbidity and mortality associated with cardiac tamponade. To our knowledge this is the first case report of JSLE presenting with both severe pericardial effusion and severe auto-immune haemolytic anaemia.

## Disclosure of interest

None declared.
